# Assessing the Current Curriculum of the Nursing and Midwifery Informatics Course at All Nursing and Midwifery Institutions in Ghana

**DOI:** 10.1177/2382120517706890

**Published:** 2017-05-01

**Authors:** Emmanuel Kusi Achampong

**Affiliations:** Department of Medical Education and IT, School of Medical Sciences, University of Cape Coast, Cape Coast, Ghana

**Keywords:** Nursing and midwifery informatics, health care

## Abstract

The use of computers in the delivery of health care has significantly improved the way health service is delivered to clients and patients in the world. Despite the importance of computing to the delivery of health service, developing countries have not greatly benefited from it. Nursing informatics has been in existence and part of academic curriculum for the past 2 decades in some advanced countries. The Ghana Nursing and Midwifery Council introduced the nursing and midwifery informatics course during the 2015/2016 academic year. This seeks to train student nurses on the relevance of computers to health care. Two separate workshops were organised to ascertain the preparedness of tutors (teachers at the nursing and midwifery training institutions) for teaching the new nursing and midwifery informatics course as well as to compare the curriculum with other international recommendations. The nursing and midwifery informatics course is taught at the first year where students have not been introduced to the nursing processes for them to appreciate the use of nursing informatics skills. It would be better if the nursing and midwifery informatics course is rather introduced during the second year second semester when students are about going for the hands-on training at the various health care institutions. Examining the course content reveals that the practical aspect within the course is very small. It is expected that more practical contents will be introduced. Tutors are not adequately prepared to teach this new course. More training is therefore needed to make tutors fully prepared to teach both the theory and practical aspects of the nursing and midwifery informatics course. It is expected that the nursing and midwifery informatics course would prepare student nurses on all nursing informatics competencies. It is essential that nurse educators incorporate the entire concept of informatics into the education of nurses.

## Introduction

Modern nursing demands constant awareness to new medications as well as new technologies. Nurses must be abreast with current research findings as well as current technologies relevant to enhance the nursing process. Nurses meet patients frequently and therefore require technology to enhance the interactions between them and the patients. The use of information and communication technologies (ICTs) to improve the safety and quality of patient care^1^ is crucial. The use of information technology (IT) could create a positive attitude and improve nursing productivity. It is important to involve nurses in the design of information systems to enhance the quality of health care.^2^ Nursing as part of the health care process, therefore, cannot remain the same at the centre of these radical transformations in IT.

Developing countries need high application of health information systems/technology as compared with developed countries because of the high disease burden as well as restricted resources and fewer health care professionals.^3^ The changing health care process requires more efficiency to ensure quality service provision to clients and patients. The Institute of Medicine (IoM) of the National Academies (USA) has stated that the application of IT in health care is one of the 5 key abilities that is required for health care professionals.^4^ Institute of Medicine has also indicated that the significance of IT in ensuring quality of health care and patient safety cannot be overemphasised.^4^ However, computer illiteracy among health professionals coupled with minimal investments in ICTs has hampered growth and use of ICT in the health sector.^5^ The introduction of ICT into the operations of health care industry without the requisite training of the health care staff and potential staff will be a recipe for disaster. The potential for the application of ICT in health care is growing because of the likelihood to improve the quality of nursing care.^6^ Thus, the inclusion of nursing informatics into the curriculum of nursing and midwifery institutions in Ghana is a step in the right direction and must be commended.

This article seeks to (1) assess the current curriculum of the nursing and midwifery informatics course for all the nursing and midwifery training institutions in Ghana, (2) compare the Ghana curriculum with International Medical Informatics Association (IMIA) and Technology Informatics Guiding Education Reform (TIGER) recommendations, and (3) find the readiness of the tutors who have not had any formal training in nursing/health informatics to teach the newly introduced nursing and midwifery informatics course.

## Nursing Informatics

Informatics is the study of how data/information is collected, analysed, stored, and disseminated. Nursing informatics integrates nursing science, computer science, and information science to manage and communicate data, information, knowledge, and wisdom in nursing practice.^7^

Nursing informatics seeks to manage the huge size of medical information. This is achieved by ensuring proper management of the medical information by promoting knowledge sharing for quality health care in nursing practice. The main objective of nursing informatics is to maximise the use of IT to ensure the provision of quality service to patients and clients. This is to ensure the safety of health care as well as increase efficiency.

Nursing informatics can be grouped into 3 major categories. These are IT, conceptual and the role of the nurse as the main actor. Information technology skills play a major role in ensuring the application of informatics skills by nurses. The conceptual aspect focuses on the need to make nurses more proactive rather than being reactive with respect to the use of informatics skills. Various roles among nurse practitioners must be identified to ensure smooth application of nursing informatics skills that have been acquired.^8^ Hence, educating students and practicing nurses on nursing informatics is important to ensure its acceptance and use.

### Nursing informatics education

According to Kaminski (2007), ‘the need to adopt a culture in nursing that promotes acceptance and use of information technology has been identified as an important parallel initiative to establishing nursing informatics competencies and educational strategies’. Consequently, plans to achieve nursing informatics competencies in the work environment must include in-service training, free access to online resources, intranet ready and online modules for teaching and learning. Training departments within health care institutions must ensure a continuing education programme for nurses in the area of nursing informatics. Nurses require regular training with the use of ICT to feel more comfortable in their work.^9^

The American Association of Colleges of Nursing and the National League for Nursing have recommended that nursing training institutions should include nursing informatics into their curriculum.^10^ This is to ensure that the application and use of IT to promote quality health care and patient safety is introduced to the would-be nurse.^10^ The TIGER has proposed some important nursing informatics competencies. They include basic computer competencies, information literacy, and information management.^1^

It is important to ensure that the nursing and midwifery informatics courses reflect current competencies reported by international organisations. It is necessary to compare the course content with the competency items as well as training recommendations by international institutions.

## Methodology

Two separate workshops were organised to ascertain the preparedness of tutors (teachers at the nursing and midwifery training institutions) for teaching the new nursing and midwifery informatics course as well as to compare the curriculum with other international recommendations.

The first workshop brought 59 tutors from 30 out of the 49 nursing and midwifery training institutions in Ghana. This workshop was organised on May 6, 2016 at the University of Cape Coast, Ghana. The workshop started from 8:00 am to 16:30 pm GMT. The focus of the workshop was to compare the nursing and midwifery informatics course curriculum with the IMIA’s recommendations for training people in informatics and other competencies listed by TIGER. At the workshop, participants were taken through IMIA requirements for all health informatics–related courses using the Recommendations of the IMIA on Education in Biomedical and Health Informatics – First Revision publication. The IMIA informatics training requirements were compared with the nursing and midwifery informatics course content. Again, the workshop also sought to find the readiness of tutors who have not had any formal training in nursing/health informatics to teach the newly introduced nursing and midwifery informatics course. This was achieved through focus group discussions with participants.

The second workshop was a follow-up one to train tutors on the nursing and midwifery informatics course topics in the curriculum. This workshop was organised from the July 27-29, 2016, at the Manna Heights Hotel, Mankessim, Ghana. The workshop brought 9 tutors from 5 nursing and midwifery training institutions. Some of the topics discussed at the workshop include history of nursing informatics, introduction to computing, data/information, nursing process, electronic health records, information security, core competencies, and nursing informatics ethics.

## Nursing and Midwifery Informatics Course

The nursing and midwifery informatics course content is quite simplified for the level of students being trained at the various nursing and midwifery institutions in Ghana. All the 59 participants indicated that they have computer laboratories in their various institutions. The number of computers available in the laboratories varied from each institution ranging from 20 to 50. The course content includes the following:

- Informatics used in nursing and midwifery practice;
- Historical roots of today’s health and nursing IT systems;
- Tools used in IT;
- Proficient use of electronic patient records in assessing a patient, making a nursing diagnosis, designing and executing a care plan, and evaluating care plan nursing interventions;
- Information technology using the Web and other research tools;
- The use of nursing and midwifery taxonomies and nursing informatics standards in Ghana;
- Vocabulary of IT terminologies;
- Contemporary issues on nursing and midwifery informatics in Ghana.

The course content was compared with the recommended knowledge and skills in nursing informatics as indicated in IMIA.^11^ Educational course content for nursing informatics should be designed to be relevant and seek to the advancement of the student.^11^ It is therefore important which year or level the nursing and midwifery informatics course is introduced to students. Presently, the nursing and midwifery informatics course is taught at the first year where students have not been introduced to the nursing processes for them to appreciate the use of nursing informatics skills. It would be better if the nursing and midwifery informatics course is rather introduced during the second year second semester when students are about going for the hands-on training at the various health care institutions.

Some topics in the nursing and midwifery informatics course content are not very clear as to the expectation of the students after going through the course. For example, IT using Web and other research tools does not specify whether the interest is in using IT for research or Web technology. The development of the course content must go through a rigorous process to ensure that the main import of the topics is stated clearly. Each course content must be compared with the expected competency required.

Nurses as IT users are required to have the basic hands-on experience in nursing informatics. Students should be introduced to basic computer literacy with expected competencies in the Microsoft Office Suite, emails, Internet, and information literacy. The focus of the training at the diploma level (3-year training) must be patient documentation which is important for managing the nursing process. Nurses should know how to access and retrieve electronic information, appreciate the use and relevance of nursing data for improving practice, and use the required application to document patient data.^12^ Nurses should be able to use administrative applications to document and review relevant patient data, critically analyse patient data, and use information management technology for patient education.^12^ Nurses should appreciate the realities of electronic communication and data management systems, explore the ethical and security issues, and solve them around data utilisation and management.^12^

Examining the course content reveals that the practical aspect within the course is very small. It is expected that any review of the course content must include how to make the course more hands-on and include many practical sessions.

International Medical Informatics Association recommends 3 credit hours per week for 14-week semester course. Notwithstanding, the nursing and midwifery informatics course was granted 2 credit hours for 14 weeks. It is expected that the credit hours for this course are increased to reflect the recommendations by IMIA.

## Faculty for Nursing Informatics

In Ghana, there is a huge gap with respect to expertise in the health/nursing informatics. Many of the tutors teaching the nursing and midwifery informatics course do not have any training in the subject area. This therefore has the potential to affect the training of these nursing students negatively. From the focus group discussions at the workshops, it was realised that about 98% of tutors who would be teaching the nursing and midwifery informatics course have not had any training in health/nursing informatics. There is therefore the need to organise training workshops for all tutors who have been teaching the course to immediately upgrade the skills and expertise in nursing informatics.

## Discussion and Conclusions

The study has considered challenges associated with the new nursing and midwifery informatics course introduced by the Ghana Nursing and Midwifery Council. Apart from the disconnection between the course content and the expected competencies, there is need to also upgrade the knowledge of tutors who are teaching the course.

There is the need to consider the competencies expected from student nurses after going through the course. This is important to ensure that the training incorporate all the necessary hands-on practicals for students to appreciate the benefits of the course. The most important support for nursing informatics is to provide tutors with educational and training opportunities. There should be continuous professional development and certificate programmes on selected topics for nursing and midwifery informatics course tutors. This will build their expertise and confidence in teaching the course.

This article has suggested recommendations for enhancing the effectiveness of the nursing and midwifery informatics training in Ghana. There is the need to link the course with the expected competencies. Various international organisations have outlined these competencies, and they are available to ensure that trained nurses can apply what they have learnt. There is need for high calibre of tutors to teach the nursing and midwifery informatics course. This can be achieved in the short to medium term by organising certificate courses and continuous professional development programmes. For the long term, there is the need to introduce bachelor’s and master’s degrees in nursing informatics in Ghana.

To promote nursing informatics in Ghana, the Nursing and Midwifery Council must be committed to ensure that nursing informatics is introduced at all levels of nursing training as well as continuing professional education and development.

For successful implementation of any health IT system, nurses should have basic knowledge about ICT, computer skills, and informatics skills. It is essential that nurse educators incorporate the entire concept of informatics into the education of nurses.

## Limitations

The study had some limitations with respect to the number of participants who participated in the workshops especially the second workshop. The number of participants for the second workshop was small. Furthermore, the number of workshops organised was small. It is expected more workshops are organised to train tutors to teach the nursing and midwifery informatics course.
